# Gold Nanoparticle-Based Fluorescent Theranostics for Real-Time Image-Guided Assessment of DNA Damage and Repair

**DOI:** 10.3390/ijms20030471

**Published:** 2019-01-22

**Authors:** Shriya S. Srinivasan, Rajesh Seenivasan, Allison Condie, Stanton L. Gerson, Yanming Wang, Clemens Burda

**Affiliations:** 1 Center for Chemical Dynamics and Nanomaterials Research, Department of Chemistry, Case Western Reserve University, 10900 Euclid Avenue, Cleveland, OH 44106, USA; shriyas@mit.edu (S.S.S.); chemra1980@gmail.com (R.S.); 2 Department of Radiology, Case Western Reserve University, 10900 Euclid Avenue, Cleveland, OH 44106, USA; agc30@case.edu; 3 Department of Hematology and Oncology, Case Comprehensive Cancer Center, Case Western Reserve University, Cleveland, OH 44106, USA; slg5@case.edu

**Keywords:** Cy7MX-loaded gold nanoparticles, AP-targeted delivery, DNA repair mechanism, chemotheranostic, real-time imaging, molecular probe

## Abstract

Chemotherapeutic dosing, is largely based on the tolerance levels of toxicity today. Molecular imaging strategies can be leveraged to quantify DNA cytotoxicity and thereby serve as a theranostic tool to improve the efficacy of treatments. Methoxyamine-modified cyanine-7 (Cy7MX) is a molecular probe which binds to apurinic/apyrimidinic (AP)-sites, inhibiting DNA-repair mechanisms implicated by cytotoxic chemotherapies. Herein, we loaded (Cy7MX) onto polyethylene glycol-coated gold nanoparticles (AuNP) to selectively and stably deliver the molecular probe intravenously to tumors. We optimized the properties of Cy7MX-loaded AuNPs using optical spectroscopy and tested the delivery mechanism and binding affinity using the DLD1 colon cancer cell line in vitro. A 10:1 ratio of Cy7MX-AuNPs demonstrated a strong AP site-specific binding and the cumulative release profile demonstrated 97% release within 12 min from a polar to a nonpolar environment. We further demonstrated targeted delivery using imaging and biodistribution studies in vivo in an xenografted mouse model. This work lays a foundation for the development of real-time molecular imaging techniques that are poised to yield quantitative measures of the efficacy and temporal profile of cytotoxic chemotherapies.

## 1. Introduction

The primary mechanism of common cytotoxic chemotherapeutic drugs involves enhancing cellular DNA damage and/or depleting DNA-repair mechanisms to prevent further cell proliferation, thereby rendering eventual necrosis of the tumor [1,2]. Following cytotoxic damage, extensive cellular repair mechanisms are activated, which lead to drug resistance and thereby further complicate the determination of a chemotherapy dosage over time. Currently, doses and dosing schedules are derived by prospective and retrospective experimental studies, which determine the putative efficacious dose, while being less than the threshold for toxicity [3,4]. Starting doses for clinical trials are determined by the dose that results in 10% lethality in animal models rather than in the direct measures of the drug’s mechanism (e.g., extent of DNA damage) [5]. Apart from the blood biomarkers or blood concentrations of the drug, there is a lack of significant biomarker metrics to assess the efficacy of treatment in an acute setting [6,7,8,9]. Thus, most chemotherapeutic treatment schedules are based on the toxicity tolerance and the lack of adverse symptoms/events. As such, methods to directly quantify and monitor chemotherapeutic action and cellular response are of great clinical need.

By leveraging specific molecular probes, imaging contrast and tissue properties, molecular imaging has the potential to provide quantitative data regarding the anatomical deposition of a drug, dynamic cellular response, tumor microenvironment, and the extent of treatment [10]. Molecular imaging can be performed repeatedly with standard controls and is non-invasive and cost-effective when compared to biopsy or radiological techniques [11]. Both in preclinical and clinical applications, molecular imaging has been used to evaluate the temporal and spatial distribution of molecular activities, including drug interactions, in biologically intact subjects. Recently developed methods enable imaging of metabolic processes, apoptosis and angiogenesis, but these provide only morphological data [12,13]. Quantitative measures of cytotoxicity are required to monitor cytotoxic drugs and titrate their doses to optimize the chemotherapeutic efficacy. 

Direct monitoring of DNA lesions is one such approach: After DNA damage by cytotoxic agents to apurinic/pyrimidinic (AP) sites, repair occurs through DNA-repair pathways, such as the base excision repair (BER), which is the most common pathway for single-base damage [14]. Therefore, monitoring AP sites provides a direct measure of the efficacy of a chemotherapeutic drug as well as the prognosis of the tumor cells. AP sites are currently measured in vitro using an aldehyde reactive probe in a chemoluminescent assay [15], however no in vivo or clinical methods exist. Previous work has used positron emission tomography (PET) imaging in consort with [^11^C]MX to bind and quantify AP sites [15]. However, the clinical usage of PET for long term and repeated monitoring may be limited by cost, resource availability, and the limits of anatomical specificity [16,17,18]. 

Recent advances have resulted in the development of numerous clinical applications and devices which leverage cellular and tissue imaging in the near infrared (NIR) range (700–900 nm) [19,20]. This is advantageous for in vivo applications, given the deeper penetration of NIR light into biological tissues that results from the decreased absorption and autofluorescence of biological tissues in the NIR region [20]. NIR fluorescence imaging has proven to be a useful diagnostic technique for clinical applications ranging from angiography and tumor detection to lymphatic mapping and revascularization delineation [21]. As compared to PET, NIR dyes can be administered repeatedly with little toxicity, are less expensive and resource-intensive, and provide high signal resolution [22]. Having strong tissue penetration, high stability, biodegradability, and minimal nonspecific absorbance, the commercially available IR-780/Cy7.5 NIR fluorescence dye has been used as a theranostic and imaging agent for tumor cells [23,24]. For example, indo-cyanine green dye-loaded nanoparticles were previously reported to localize in folate-overexpressed tumors in vivo and in vitro. IR-780 has been used as highly selective tumor cell imaging and targeting agent in the NIR region [25,26]. 

Given the recent advances in molecular probe development and NIR imaging, we assembled a molecular probe system which is active in the NIR spectrum: methoxyamine-modified cyanine-7 (Cy7MX). [^14^C]methoxyamine [MX] binds directly to AP sites in proportion to the extent of the DNA damage (via BER or other DNA-repair mechanisms) and can be used as a quantitative indicator for the damage induced by a chemotherapy [23,27]. In order to deliver the nonpolar Cy7MX probe in the blood, we need a highly stable carrier system that can carry the molecular probe effectively in the blood and deliver selectively to tumors. Among others, gold nanoparticles (AuNP) offer an ideal carrier system for loading and delivering the molecular probes, as the shape, size, and surface structure are optimal for small molecule delivery [28]. When coated with polyethylene glycol (PEG), AuNPs provide a stable amphiphilic surface for lipophilic chemotherapeutic agents [29]. PEGylated AuNPs have demonstrated highly efficient drug delivery into tumors by avoiding clearance via the reticuloendothelial system (RES), and deposit within minutes of injection [30,31]. Exploiting the enhanced permeability and retention (EPR) effect, AuNPs are able to selectively deposit cargo in tumors and remain bioinert until they are excreted [32,33]. We therefore employed a PEGylated AuNP carrier system to facilitate the systemic transport of the molecular probe in the blood environment and deliver the probe to tumor cells. Imaging of this system in vivo enables a real-time analysis of cytotoxicity.

In this paper, we present a molecular imaging technique to directly measure the cytotoxicity induced by chemotherapeutic agents as a function of AP sites. We have designed a Cy7MX-loaded PEGylated AuNPs-based drug-carrier platform to selectively deliver and release a unique NIR molecular probe that binds directly to AP sites of DNA damage. In vitro DLD1 colon cancer cell line experiments explore the specificity of the molecular probe by profiling cellular uptake during chemotherapy. Further, in vivo mouse models are used to determine the selective delivery and fluorescent readout of our probe. This study aims to lay the foundation for a Cy7MX-loaded PEGylated AuNP system that can provide a quantitative measure of the chemotherapeutic drug’s effect, uptake kinetics and cytotoxic chemotherapy efficacy.

## 2. Results

### 2.1. UV-Vis Characterization of Molecular Probe and AuNPs

**Figure 1:**
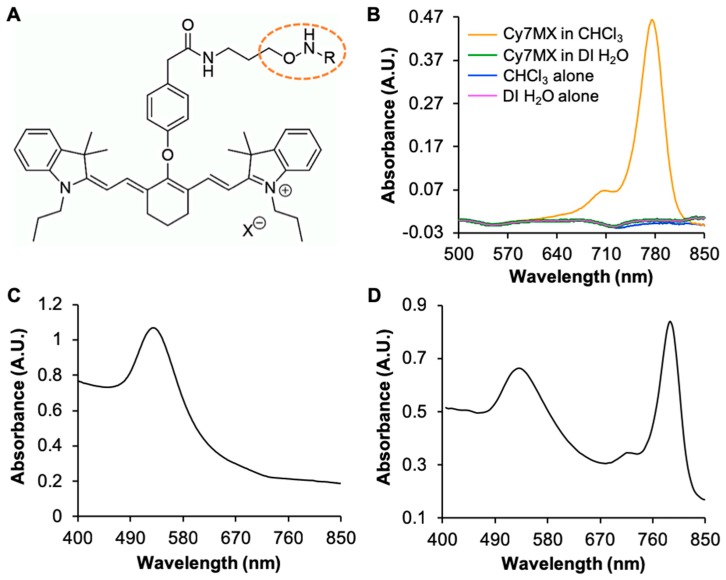
(**A**) Structure of Cy7MX exhibiting methoxyamine (MX) addition to Cy7 highlighted in dashed orange circle. (**B**) Absorbance spectra of Cy7MX in different solvents; CHCl_3_ (blue) and DI H_2_O (pink) are negative controls, Cy7MX in CHCl_3_ (orange) and Cy7MX in DI H_2_O (green). The Cy7MX exhibited absorbance at 780 nm only when in CHCl_3_ (orange), demonstrating its hydrophobicity. (**C**) Absorbance spectrum of PEGylated AuNPs in CHCl_3_ with peak absorbance at 532 nm. (**D**) Cy7MX-AuNPs in CHCl_3_ exhibited two peaks: 782 nm for Cy7MX and 539 nm for AuNPs at a ratio of 10:1 (10 Cy7MX dye molecules per AuNPs).

Cy7MX (Figure 1A) was first characterized in an array of solvents to determine its aptitude for delivery through the blood. The absorbance spectra of DI H_2_O, chloroform (CHCl_3_), and Cy7MX in CHCl_3_ and DI H_2_O is shown in Figure 1B. Cy7MX exhibits a strong peak (λ_max_ = 780 nm) in the NIR range when in the nonpolar CHCl_3_ medium, but not in H_2_O. This indicated that Cy7MX is highly nonpolar in nature, impeding it’s naked delivery intravenously. Thus, we synthesized AuNPs to serve as a carrier for the molecular probe.

The Brust–Schiffrin method was used to synthesize the AuNPs. Strong absorbance at 532 nm was observed (Figure 1C) which indicated the disperse formation of AuNPs and no aggregation or precipitation in the sample. Dynamic laser scattering (DLS) was used to characterize the size and polydispersity of the synthesized AuNPs. The DLS data indicated that the average size of AuNPs was 26.4 ± 6.5 nm with a polydispersity of 0.133. These dimensions were in the range determined to be ideal for this application as it yields long circulation times and a simultaneous permeation of the leaky vasculature characteristic in tumors [31]. We then PEGylated the AuNPs to grant biocompatibility, stealth nature, favorable stability, and amphiphilic properties which, when combined, yield targeted delivery capabilities [31,34]. 5K PEG was chosen based on prior studies which optimized the PEGylation specifically for cancer uptake [28]. TEM was performed to assess the size and dispersion of the particles as well as confirm the ligand exchange (Figure S1). We chose to use a thick layer of PEG to incorporate the nonpolar Cy7MX drug as a molecular probe and shield it from the nonpolar environment of the blood. 

### 2.2. Cy7MX-AuNPs Loading Profile

Cy7MX was loaded onto the AuNPs at a 10:1 ratio and characterized during and after the loading process. Cy7MX, which is hydrophobic, localized within the PEG corona and adsorbed to the surface of the AuNPs given the amphiphilic nature of the PEG corona. In Figure 1D, the UV-Vis absorbance spectrum of 10:1 loaded Cy7MX-AuNPs in CHCl_3_ demonstrates well distinguishable peaks at 539 nm and 782 nm, indicating that both the molecular probe, Cy7MX, and carrier AuNPs were present in the final product at the desired 10:1 ratio. The synthesized product was stable and did not decompose for at least 3 weeks at 25 °C and 4 °C. While Cy7MX is sensitive to light and can photo-bleach rapidly (20 min in ambient light), it was shielded once loaded onto the AuNPs and retained its fluorescence for longer time periods.

The Cy7MX-AuNPs were analyzed in fetal bovine serum and phosphate buffered saline (PBS) to simulate the physiological conditions of blood. This yielded insignificant differences in the resulting spectra which indicated viable stability in biological tonicity and osmolality. We additionally tested the Cy7MX-AuNPs in acidic and basic conditions using media ranging from pH 5 to 9 to account for any unexpected behavior that may result from microenvironmental changes in the blood. No significant differences were observed in the absorbance spectra of the Cy7MX-AuNPs in these media.

**Figure 2:**
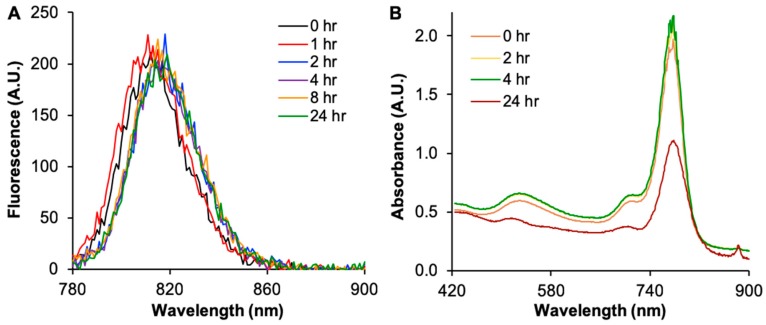
(**A**) Fluorescence spectra of Cy7MX-AuNPs during the dye loading process at 0, 1, 2, 4, 8, and 24 h. The particles were excited at 770 nm and the emission was measured at 775 nm. Cy7MX dye loading occurred in CHCl_3_ with a 10:1 (Cy7MX:AuNPs) ratio. The upward trend of the curve over time reflects the movement of the Cy7MX probe into the PEG corona of the AuNPs yielding a weaker signal in response to 770 nm excitation. (**B**) Absorbance spectra of Cy7MX-AuNPs at 0, 2, 4, and 24 h during the Cy7MX dye loading process demonstrates a significant decrease (50%) at 24 h, which reflects the adsorption of the Cy7MX to the AuNPs surface.

The absorbance and fluorescence of the synthesis solution was monitored during the Cy7MX dye-loading process (Figure 2) to detect the quantity of Cy7MX present in solution. Over the course of 24 h, we observed a shift in the fluorescence peak to higher wavelengths in the range from 813 to 820 nm, which reflected the decrease of Cy7MX in the solution and the concurrent localization of Cy7MX in the hydrophobic PEG corona of the AuNPs (Figure 2A). Simultaneously, there was a significant decrease in absorbance at the 24 h time point, indicating that the Cy7MX was effectively loaded onto the PEGylated AuNPs surface (Figure 2B). The peak heights at 532 nm and 780 nm demonstrated the final ratio yielded from the synthesis.

### 2.3. Biphasic Release Profile

**Figure 3:**
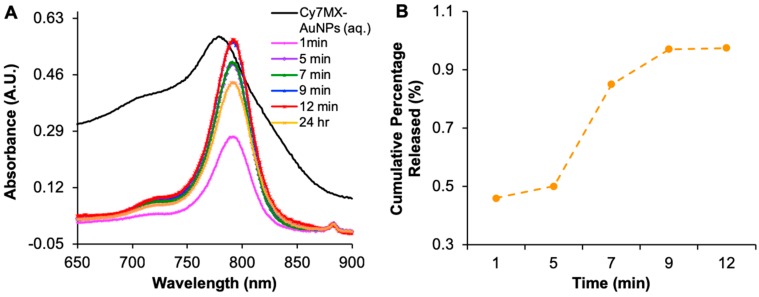
(**A**) Absorbance spectra of nonpolar layer at 1, 5, 7, 8, 12 min and 24 h capturing the release of Cy7MX (characterized by 780 nm peak) into a nonpolar layer from Cy7MX-AuNPs present in aqueous media. Growth and sharpening of peak from 1–12 min demonstrates rapid release of Cy7MX into the nonpolar layer. (**B**) Cumulative release profile of Cy7MX-AuNPs from aqueous to nonpolar environment demonstrates 97% release at 1, 5, 7, 9, and 12 min.

A biphasic release study was carried out to characterize the release of Cy7MX from the AuNP into nonpolar environments. First, Cy7MX-loaded AuNPs were placed in aqueous media (representing the aqueous environment of the blood) and then CHCl_3_ was layered on top as the nonpolar layer (representing the hydrophobic environment of the cellular membranes). The absorbance of both layers was then monitored over time (Figure 3A).

As expected, we observed an increase in the absorbance of the nonpolar layer over time, resulting from the hydrophobic effect driving Cy7MX from the AuNPs surface into the nonpolar layer. The absorbance data indicated that Cy7MX releases rapidly in the first 9 min and gradually reaches an equilibrium at 12 min. The change in morphology and stabilization of the 780 nm peak at 24 h reflects the decomposition of Cy7MX over time. It is important to note that the absorbance spectrum also shifts slightly leftward, which is characteristic of pure Cy7MX and opposed to its fluorescent fingerprint when loaded on the AuNPs. The cumulative release profile is calculated as a ratio of dye in the nonpolar layer based on the starting concentration of the aqueous layer. This indicated a 97% release rate at 12 min (Figure 3B). Throughout, Cy7MX concentration decreased in the aqueous layer while the concentration of the AuNPs remained constant. This suggests that the PEGylated AuNPs remained in the aqueous layer and only allowed the Cy7MX to be released into the nonpolar layer for which it had greater affinity.

### 2.4. In Vitro Uptake of AuNPs and Cy7MX

Given the desirable loading and release profiles demonstrated by the Cy7MX-AuNPs for a practical application and clinical settings, we tested the Cy7MX-AuNPs interaction with cells in culture media to determine the uptake and release behavior in a cellular environment. Our molecular probe-carrier system was designed to release only the Cy7MX probe into cells and not have an unnecessary cellular uptake of any AuNPs, which could lead to cell toxicity. To confirm this, we compared DLD1 colon cancer cells incubated with AuNPs or PBS (1x, negative control) by monitoring the absorbance and atomic absorption spectra (AAS) of the lysate. Cellular membranes were perforated in a subset of cells using sodium dodecyl sulfate (SDS) to serve as a positive control, which would enable AuNP entry into the cell. 

**Figure 4:**
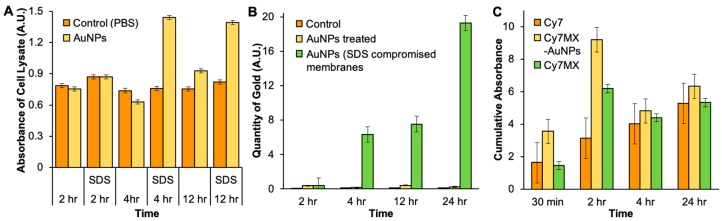
(**A**) Absorbance of AuNPs was monitored in DLD1 colon cancer cells incubated with AuNPs (yellow) or a control PBS (orange) solution at 2, 4, and 12 h. A subset of cells, serving as a positive control, were treated with SDS and resulted in perforated cell membranes allowing AuNP entry. At each time point, no significant difference between the AuNPs treated and untreated (control) groups in the normal cells was observed. However, in groups with perforated cell membranes (SDS), AuNPs entered the cells. (**B**) Atomic absorption spectroscopy of the cells treated with PBS as control (orange), AuNPs (yellow) and AuNPs-SDS (green) demonstrates AuNP entry only when membranes were compromised. (**C**) Cells were treated with control Cy7 (orange), Cy7MX-AuNPs (yellow) or Cy7MX (green) to characterize the delivery and cellular uptake properties. The integrated absorbance measured from 650–800 nm of cell lysates at the 30 min, 2, 4, and 24 h time points indicates that Cy7MX was delivered at a significantly greater rate than the control when carried by AuNPs. Both absorbance spectroscopy and AAS results were statistically significant at the *p* = 0.05 level (student’s *t*-test).

Figure 4 demonstrates insignificant differences between the AuNPs concentrations as measured by absorbance spectroscopy (Figure 4A) or particle counts (Figure 4B) in control and treated cells at all time points. While AuNPs significantly accumulated in cells with perforated membranes (treated with SDS) after the fourth hour, they did not enter the cells with intact membranes. Both absorbance spectroscopy and AAS results were statistically significant at the *p* = 0.05 level (student’s *t*-test). Variance in the absorbance of the groups treated with PBS (see orange color) over time was due to the changing color of the medium. These results confirmed that the AuNPs used in this study were not taken up by cancer cells and could be used with minimal concerns of intracellular toxicity. Further, systemic clearance of AuNPs when administered intravenously is less than 12 h, which enables their delivery and clearing from the system before considerable cellular deposition may occur [6].

To characterize the effect of the NP on the uptake rate of Cy7MX by cells, we performed an in vitro study in which DLD1 colon cancer cells were treated with either Cy7, Cy7MX, or Cy7MX-AuNPs (Figure 4C). At each time point, cells were collected, washed, lysed, and the concentration of Cy7MX was measured. Figure 4C demonstrates that Cy7MX was taken up by cells at a rate significantly greater when delivered using the AuNP carrier, as compared to Cy7MX alone at 30 min and 2 h. This is facilitated by the hydrophobic effect: Cy7MX is adsorbed to the surface of the PEGylated AuNPs and can be easily dispersed in the aqueous cell culture media. Once the AuNPs make contact with the cell surface, the amphiphilic nature of the cellular membranes and the NPs draws the probe into the cell through hydrophobic interactions. Between 30 min and 2 h, we observed a large increase in the uptake followed by a gradual decrease. This decrease can be attributed to Cy7MX decomposing due to contact with light and other molecules in the media. Because these cells were not treated with chemotherapy, there was no preferential binding affinity for Cy7MX over Cy7. Thus, for most time points, the uptake of Cy7MX was not significantly different from Cy7 and represents the baseline rate of uptake.

**Figure 5:**
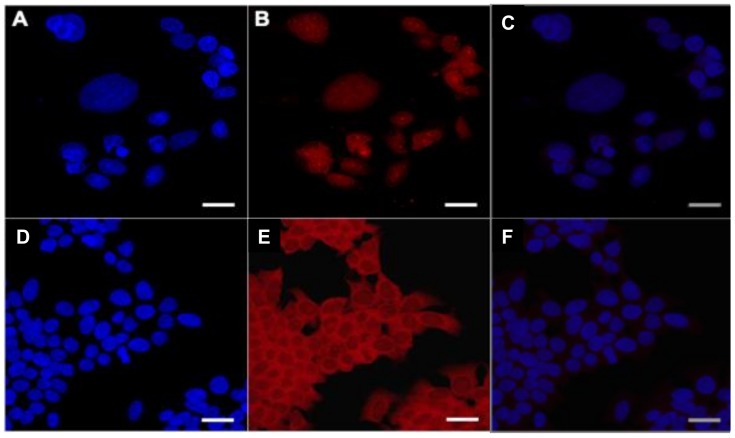
DLD1 colon cancer cells treated with chemotherapy are (**A**) stained with DAPI to visualize the nuclei and (**B**) imaged for Cy7MX. Significant overlap in the nuclear region is visualized indicating uptake of Cy7MX in the nucleus. (**C**) Merged images from A and B demonstrate low presence of the probe in the cytoplasm, but a high accumulation in the nucleus. (**D**) Cells not treated with chemotherapy were stained with DAPI to visualize the nuclei and (**E**) imaged for Cy7MX, demonstrating greater cytoplasmic uptake and relative exclusion from nuclei. Scale bar = 6 µm. (**F**) Merged images from D and E demonstrate little uptake into the nucleus and a high proportion of probe existent in the cytoplasm.

In a separate assay, we treated DLD1 colon cancer cells with chemotherapy or PBS (control) and incubated them with the Cy7MX-AuNPs to identify whether the Cy7MX localized in the nucleus to a greater degree once cytotoxic damage had occurred. We performed confocal microscopy of cells treated with and without chemotherapy and visualized the nuclei by staining with 4′,6-diamidino-2-phenylindole (DAPI). The obtained images are shown in Figure 5A–F. Quantification of the confocal microscopy images yielded a significant difference between the ratio of Cy7MX found in the nucleus versus cytoplasm between the treatment groups. A two-sample *t*-test demonstrated that these results were significant at the *p* = 0.015 level, indicating that Cy7MX localized in cell nuclei in a cytotoxicity dependent manner (Figure 5B,E). In cells that were not treated with chemotherapy, containing the baseline level of AP sites, Cy7MX was still taken into the cell, but did not have preferential uptake in the nuclei, as seen in Figure 5B. In fact, the images revealed a distinctive exclusion from the nuclei in these cells. These in vitro experiments demonstrated stable loading and release profiles, competitive uptake and localization into tumor cell nuclei for the Cy7MX platform, and laid the foundation for in vivo experimentation.

### 2.5. Xenografted Tumors Treated with Cy7MX-AuNPs

**Figure 6:**
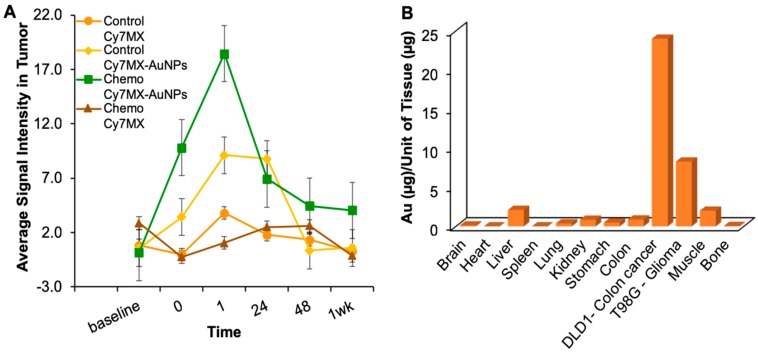
(**A**) Mice xenografted with tumor cells were administered one of four treatments: (1) Saline control and Cy7MX (orange), (2) saline control and Cy7MX-AuNPs (yellow), (3) chemotherapy and Cy7MX (brown), and (4) chemotherapy and Cy7MX-AuNPs (green). Mice were fluorescently imaged at the time of administration, 1, 24 and 48 h, and 1 week after administration and signal intensity in the tumor region was quantified. Uptake of Cy7MX-AuNPs was significantly greater (*p* < 0.01) at the administration, 1 h and 24 h time points when compared to Cy7MX delivered without a carrier. Furthermore, a comparison between the groups treated with Cy7MX-AuNPs demonstrated significantly increased binding quantities at the 1 h time point in the group treated with chemotherapy as opposed to control. (**B**) Average biodistribution of AuNPs in harvested tissues from xenografted mice treated with Cy7MX-AuNPs demonstrated significantly higher accumulation in xenografted tumors when compared to all other tissues. Muscle and liver were the only other tissues with considerable accumulation above the baseline.

To assess the effectiveness of the platform in vivo, mice (*n* = 20) were xenografted with DLD1 colon cancer cells on either flank. After two weeks, mice were either treated with chemotherapy (5-Fluorodeoxyuridine at a dosage of 0.2 mg of drug per gram of mouse) (*n* = 10) or 1x PBS (*n* = 10) every three days. After three doses, Cy7MX (*n* = 5) or Cy7MX-AuNPs (*n* = 5) was intravenously administered and fluorescence imaging was performed with deep red excitation and emission filters (750–950 nm). Mice treated with chemotherapy were expected to have a greater quantity of AP sites in DNA and consequently, have higher a signal intensity resulting from greater Cy7MX binding. Additionally, it was expected that the probe would have a greater circulation, penetration, and accumulation when delivered using the AuNPs carrier system. Figure 6A demonstrates the measured signal intensity in the flank regions containing the xenografted tumor for each group over the course of one week. As predicted, animals treated with chemotherapy exhibited signals significantly higher (*p* < 0.01, student’s *t*-test) than the untreated controls. Figure S2 demonstrates the uptake profile of the Cy7MX and Cy7MX delivered on the AuNP carrier in representative animals treated with chemotherapy. Additionally, delivery through the AuNP carrier facilitated circulation for longer periods of time as seen by the heightened fluorescence signal in Figure S2 for the animals treated with Cy7MX-AuNPs. Mice in which the AuNPs carried the Cy7MX showed significantly greater localization of the probe, especially 1 h after the injection. At 48 h, we observed 70% clearance of the probe from all tissues, which is consistent with standard clearance rates for hydrophobic drugs [35]. 

### 2.6. Biodistribution Following Systemic Administration of Cy7MX-AuNPs

After one week, organs from all animals were harvested and tissues were processed and analyzed with AAS to detect the quantity of gold (Au) present in each tissue. This analysis indicates the efficacious delivery and distribution of the AuNPs to the target organs and is predictive of potential toxicity sites due to unwanted accumulation of the AuNPs. Figure 6B demonstrates the measured values of Au per gram of harvested tissue in animals administered the Cy7MX-AuNPs. Tissues from animals administered only Cy7MX resulted in no Au AAS signal and served as a negative control. Quantities found in the glioma and colon cancer xenografts were more than two-fold higher than any other tissue. The colon cancer tumor especially had a significantly higher quantity of accumulated Au than other tissues (*p* < 0.05, student’s *t*-test). Liver tissue also demonstrated some accumulation, which was expected given its clearing function for the body [35]. The lack of significant Au quantities in other tissues is evidence of the strong targeting functionality of the AuNPs and suggests that it is a suitable carrier platform for this application.

## 3. Discussion

Since AP sites are key intermediates in the BER pathway, their quantitative and dynamic measurement in cellular DNA is crucial for the efficacy evaluation of therapeutic treatments. Currently, the assessment of AP sites can only be achieved in vitro using extracted DNA from tumor tissues. However, this is a relative measure based on chemiluminescence using an aldehyde reactive probe (ARP). ARP is an invasive assay and cannot be used to directly monitor AP-site content in tumors. The direct imaging and quantitative assessment of AP sites in vivo in real-time will provide a platform technology for efficacy evaluation of a variety of DNA-targeted chemotherapies—any that produce AP sites and invoke BER. Understanding the dynamic of AP site formation and repair will allow physicians and researchers to determine optimal dose strategies of single and combination treatment schedules. Furthermore, the direct imaging of AP sites will help to determine the optimal dose schedule to potentiate drug administration based on persistence of AP sites. 

In this study, we developed a NIR-based imaging technique that can directly measure the cytotoxicity induced by chemotherapeutic agents as a function of AP sites. Using a Cy7MX-loaded PEGylated AuNPs-based drug-carrier platform, we selectively delivered and released a unique NIR molecular probe in the animal models that bound directly to AP sites of DNA damage in vivo. NIR imaging is cost-efficient, easy to operate, and has the advantage of multichannel imaging. Further, heptamethine cyanine dyes have been used extensively in NIR fluorescent imaging as contrast agents for tumor imaging. For these reasons, we developed a cyanine-based NIR probe, Cy7MX, which exhibits promising properties of binding to AP sites. Because inherent NIR fluorescence of Cy7MX can penetrate the skin, the subsequent NIR fluorescent scan in flank xerograph tumor models would thus allow the detection and quantification of AP site formation in real-time in the tumor tissues directly under the skin.

The salient objectives of this study were to (1) design and prepare a molecular probe that specifically bound to AP sites and (2) study the efficacy when delivered using a PEGylated AuNPs carrier platform tuned for delivery through blood [28,36]. We applied a simple, but elegant mechanism relying on the interactions of the hydrophobic molecular probe with the biological milieu: We loaded Cy7MX onto AuNPs through successful shielding of the hydrophobic Cy7MX by the amphiphilic PEG. The NPs circulated the blood stream and selectively delivered Cy7MX to tumorous tissues, when the AuNPs achieved contact with cellular membranes. This mechanism works because of the combination of the mechanical properties of the Au NPs and the polarity effects between Cy7MX, the PEG coating, and the cellular surface. Spectroscopic studies were used to characterize the CY7MX dye and AuNPs platform and study the loading and release behavior in nonpolar and polar media, which forms the basis for the probe-carrier delivery mechanism. Further, in vitro cell culture tests demonstrated targeted binding of Cy7MX to AP sites and specific localization in the nuclei. Finally, delivery to xenografted tumors in a mouse model exhibited highly targeted delivery to tumor sites and signal intensities correlating to chemotherapeutic treatment. It should be noted that once Cy7MX binds to AP sites, it prevents further repair of DNA, which leads to eventual cell death and tissue necrosis, an extra feature of the system. This study establishes Cy7MX-AuNPs to be a highly viable platform for a molecular imaging technique to quantify cytotoxic chemotherapy through fluorescence imaging in the NIR region. To date, there have been few similar combined approaches that strategically leverage the properties of nanoparticles with chemo-diagnostic molecular probes.

In its envisioned clinical implementation, an oncologist would administer a chemotherapeutic drug, followed by our molecular probe. Periodic imaging in the hours and days following treatment would enable the clinician to ascertain the efficacy and potential development of resistance to the chemotherapy. Using this data, the clinician could accordingly titrate the dosage in order to minimize toxicity and increase efficacy in a data-driven fashion. Currently, the clinical correlate is a blood test for a biomarker that is an indirect readout of the chemotherapeutic effect. Blood tests not only require invasive, repetitive draws, but also provide a readout only once a pathology lab completes the appropriate assay—which could take hours or days. In contrast, this system provides a molecular-scale real-time reporter of the success of chemotherapy in a format that is non-invasive, repetitive, non-destructive, cost-effective, and in real-time [11]. 

## 4. Materials and Methods

### 4.1. Synthesis of DDA-Coated AuNPs

AuNPs were selected to be the carrier platform to load the Cy7MX probe in this study. AuNPs were coated with dodecylamine (DDA) through the Brust–Schiffrin method to provide a monodisperse solution and prevent aggregation according to a published synthesis approach [30]. Briefly, 0.25 mM of tetraoctylammonium bromide (294136, Sigma–Aldrich, St. Louis, MO, USA) and 0.6 mM DDA (325163, Sigma–Aldrich) were dissolved in 5 mL toluene (244511, Sigma–Aldrich). Then, 0.53 mmol gold(III) chloride trihydrate; HAuCl_4_·3H_2_O (520918, Sigma–Aldrich) solution (30% in HCl solution) was added into the above mixture and allowed to stir for 2 h at 25 °C. Next, 0.25 mM of aqueous NaBH_4_ (452904, Sigma–Aldrich) was added to the organic phase and stirred for 2 h at 45 °C. The obtained nanoparticles were precipitated in ethanol (24194, Sigma–Aldrich) and redispersed in 3 mL of CHCl_3_ (C298-500, Fisher Scientific, Pittsburgh, PA, USA).

### 4.2. PEGylation of DDA Coated AuNPs

A layer of PEG was added to the AuNPs to confer desirable stability, shielding, and delivery benefits in biological media [30]. Ligand exchange was performed with alpha-methoxy-omega-mercapto poly(ethylene glycol) (PEG, MW = 5000) (Meo-PEG-SH) (Laysan Bio, Arab, AL, USA) ligands. UV-visible spectroscopy (Cary Bio50, Varian spectrophotometer, Santa Clara, CA, USA) was used to determine the concentration of the AuNPs. A 1:500 ratio of AuNPs:PEG ligands was stirred in CHCl_3_ for 48 h at 25 °C. This ratio was determined to be optimal for the nanoparticles as it provides maximum stability and dispersity [28]. The CHCl_3_ was then evaporated and the PEGylated AuNPs were dissolved in distilled water. Centrifugation (14,000 rpm, 5 min) was performed twice to remove unattached PEG ligands. Dynamic laser scattering (DLS) was performed to determine the size, morphology, and dispersity of the particles. Particles were analyzed in water. Transmission electron microscopy (TEM) was also performed to confirm ligand exchange and visualize the dispersion of particles. 

### 4.3. Cy7MX Dye Loading on PEGylated AuNPs

First, Cy7MX was synthesized as previously reported in Condie et al., 2015 [37]. Briefly, the cyanine-7 dye (425311, Sigma–Aldrich) was coupled with tert-butyl 3-(2-(4-hydroxyphenyl) acetamido)propoxycarbamate (prepared according to Salisbury et al., 2002 [38]) in the presence of sodium hydride (223441, Sigma–Aldrich) to give the substitution product, which was subsequently deprotected with trifluoroacetic acid (302031, Sigma–Aldrich) to yield Cy7MX with a 19% overall yield. Cy7MX dye was then loaded onto the AuNPs to provide a shielding effect from hydrophilic media and an eventual release into hydrophobic media. UV-visible spectroscopy was used to determine the concentration of the PEGylated AuNPs. A 1:10 ratio of AuNPs:Cy7MX was stirred in CHCl_3_ for two days at 25 °C. The fluorescence (emission at 780 nm) and absorption spectra of the sample were measured at 1, 2, 4, 8 and 24 h. Absorbance and fluorescence spectra (excitation at 780 nm) of the dye in CHCl_3_ (C607SK-1, Fischer Scientific) were obtained as a control for comparison against the dye when loaded on PEGylated AuNPs. The organic phase was evaporated and the Cy7MX dye-loaded PEGylated AuNPs were redissolved in DI H_2_O under sonication for 30 min.

### 4.4. DLS Characterization

Because the size of the AuNPs platform can greatly influence the delivery properties of the system, DLS was used to determine the hydrodynamic size of the AuNPs and ensure a normal distribution. Particles were analyzed in water. A BI-200SM laser light scattering goniometer with a BI 9000AT autocorrelator from Brookhaven Instruments Corp was used to perform DLS. Each sample was measured in triplicate for one minute each at a detection angle of 90° from a 200 μm pinhole. The dust cutoff was set to 5000. The output measurement was the lognormal mean number averaged diameter.

### 4.5. Loading and Stability of Cy7MX

UV-visible spectroscopy was performed to detect a monodisperse sample of AuNPs, which is indicated by an absorbance spectrum at 532 nm, whereas aggregation in the sample is indicated by a red shift above 532 nm. For each measurement, 2 mL of sample was placed in a quartz cuvette and placed in the spectrophotometer at room temperature for both types of spectroscopy. The scans were performed at the medium speed setting. The spectra of AuNPs before, during, and after loading of Cy7MX were obtained and analyzed to yield information regarding the loading behavior. Additionally, the spectra of Cy7MX in aqueous (DI H_2_O) and organic (CHCl_3_) media were obtained. A Varian Eclipse Fluorescence Spectrophotometer was used for fluorescence measurements at an excitation of 780 nm with a slit size of 2.5 mm, scanned at medium speed. The spectra of Cy7MX-AuNPs in different solvents were compared by absorbance and fluorescence to check the stability of the particles in conditions that were isotonic, acidic, and isosmotic relative to that of biological systems.

### 4.6. Biphasic Release Study

To gauge the time profile and release characteristics of the Cy7MX dye from the surface of the AuNPs, a biphasic release study was carried out. 1 mL of Cy7MX-AuNPs in DI H_2_O was placed in a cuvette. 2 mL of CHCl_3_ were added to the cuvette and the phase-separated system was stirred for 24 h. Absorbance of the layers was measured from 200 to 800 nm periodically for 24 h.

### 4.7. AuNPs Uptake

To quantify the AuNPs transport into cells, a cellular uptake study was carried out on DLD1 colon cancer cells (ATCC #CCL-221) obtained from the laboratory of Sanford Markowitz at Case Western Reserve University. DLD1 WT cells [37] were seeded into 6-well plates at a seeding density of 0.3 × 10^6^ cells/mL and grown to 70% confluence in cell culture growth media. Cells were treated with 2 mL of either 0.01 µM PEGylated AuNPs or PBS (control group) in media. An identical group was treated with 5% SDS in the media to perforate the cell membrane and serve as a negative control. At 2, 4, 12, and 24 h, the medium was removed, and the cells were washed with 2 mL of PBS three times. The cells were then harvested from the substrate with 2% typsin-EDTA. The absorbance of the lysate was measured and the wavelengths of 510–550 nm were integrated to determine the concentration. The lysates were further analyzed by graphite furnace atomic absorption spectroscopy (GFAAS) in a GTA-110 with a programmable auto-sampler (Varian, Inc., Palo Alto, CA, USA). The wavelength of the hollow cathode gold (Au) lamp was 242.8 nm and values for the concentration of Au in the samples were calibrated by a series of Au standard solutions.

### 4.8. Cy7MX Uptake

To identify the quantities of the Cy7MX dye in PEGylated AuNPs and the time profiles of uptake by cells, an uptake study was carried out. The cultured cells were treated with 2 mL of either 0.1 µM Cy7MX-AuNPs or 0.1 µM Cy7MX or PBS in media. At 30 min, 2, 4, and 24 h, the media was removed and the cells were washed with 2 mL of 1× PBS three times. The cells were harvested from the substrate using cell scrapers. The absorbance of the lysate was measured and integrated from 750–800 nm (Cy7MX absorbance peaks at 780 nm).

### 4.9. Cy7MX Localization

To ensure the localization of Cy7MX in nuclei, 20,000 DLD1 cells were plated on glass coverslips in 6-well cell culture plates. DLD1 cells were incubated in 2 mL of media for 24 h. 2 mL of 1 mM 5-florouracil (F6627, Sigma–Aldrich) diluted in media or 2 mL of DMEM media were added to two groups of cells. At 96 h, media was removed, and the cells were washed with 1× PBS twice. Cells were then fixed with 2 mL of methanol at −20 °C for 10 min. Uracil DNA glycosylase (UDG) treatments in UDG reaction buffer—either 100 or 250 units—were added to each sample and incubated overnight. Cells were then washed with 1× PBS and incubated with a 25 µM Cy7MX probe for one hour while stirring. The samples were then rinsed with 1x PBS, stained with 4′,6-diamidino-2-phenylindole (DAPI), and soft mounted onto slides. Cells were imaged with a Leica STP 600 confocal microscope under 80x magnification. Excitation was performed at 405 nm and 633 nm with detectors measuring DAPI from 410–480 nm (32% gain) and Cy7MX from 650–680 nm (41% gain). Images were analyzed with ImageJ.

### 4.10. In Vivo Testing of Cy7MX Uptake

In vivo imaging experiments on colon cancer xenografted NCR nude mice were carried out at Case Western Reserve University in the Case Center for Imaging Research in Cleveland, Ohio under the IUCAC protocol (ID# 2014-0067) approved by the Case Western Reserve Institutional Animal Care and Use Committee Office on 16 March 2016. Twenty 8-week old NRC nude mice (6–8 weeks of age) were fed a Teklad 2018 S special alfalfa-free diet for two weeks to reduce auto fluorescence. DLD1 colon cancer cells and T98G glioma cells were cultured in cell growth media and were used at passage 12 and 8, respectively. At 90% confluence, cells were harvested, and each mouse was injected with 3 million cells per tumor site—glioma on the right flank and colon cancer on the left. After two weeks, the tumors had grown to approximately a centimeter in width. Half of the mice were treated with the chemotherapeutic drug 5-Fluorodeoxyuridine (5-Fdu) at a dosage of 0.2 mg of drug per gram of mouse every three days. PBS (806544, Sigma–Aldrich, St. Louis, MO, USA) was administered to the other half as placebo. After 3 doses, the mice were injected intravenously with either 100 µM Cy7MX or 100 µM Cy7MX–10 µM AuNPs. The Cy7MX concentration was 5 nmol per 25 g of mouse. Chemotherapy continued during the duration of imaging and the mice were kept in a normal ambient light environment.

Using a Maestro in vivo fluorescence imaging system (Cambridge Research and Instrumentation, Inc., Woburn, MA, USA), black and white photographs as well as fluorescence images were obtained before injection, at injection, and 1, 24, 48 h, and 1 week after injection. Deep red (750–950 nm) excitation and emission filters were used. Images were obtained with 3 s exposure. The multispectral images were separated into their component spectra (Cy7MX, auto fluorescence, and background). After removing the background and auto fluorescence, the signal intensity in tumor regions was quantified. The tumor regions were identified to be regions of interest from the black and white images and overlaid onto the fluorescence images to quantify signal.

### 4.11. Biodistribution

After one week, the mice were sacrificed and organs including the heart, liver, brain, tumors, and kidneys were isolated and harvested. All tissues were digested with 70% nitric acid (HNO_3_) (438073, Sigma–Aldrich) at 35 °C for 48 h. Digested samples were diluted in 2.5 mL of HNO_3_ and analyzed with a GFAAS in a GTA-110 with a programmable auto-sampler (Varian, Inc., Palo Alto, CA, USA). The cathode Au lamp had a wavelength of 242.8 nm. A set of standards was used to create a calibration curve of known values. Using this calibration as a guideline, the concentrations of Au in the organ samples were found.

## 5. Conclusions

In this work, we hypothesized that a molecular probe on a AuNP carrier platform would enable the targeted delivery of a highly selective molecular probe in blood. Our results demonstrate that the Cy7MX molecular probe can be stably loaded onto AuNPs, specifically bound to AP sites, selectively delivered to tumors, independently released to cells, and taken up by nuclei undergoing chemotherapy. Our in vivo experimentation validated the mechanism and efficacy of the system. Furthermore, our in vivo experimentation in xenografted mice additionally demonstrated no adverse toxicity or unintended deposition of AuNPs. Future work should focus on increasing the binding specificity of Cy7MX to AP in order to increase the specificity of the assay. The translation of this technique to a clinical modality would require the customization of the probe release and cellular uptake dynamics of each chemotherapy. Further, a correlation between signal intensity and dosage would have to be developed and calibrated to subject weight and tissue composition. Molecules that improve uptake and cellular binding, and avoidance of the immune system could be explored as modifications to refine the system. This study serves as a proof of concept for a data-driven methodology that will enable clinicians to determine more accurate doses, time schedules, and gather information regarding a drug’s specific interactions in the tumor microenvironment. This will not only have applications in oncological clinical medicine by potentially yielding improved treatment and patient safety, but could also be implemented in the pharmaceutical industry to aid drug discovery and design.

## Supplementary Materials

The following are available online at http://www.mdpi.com/1422-0067/20/3/471/s1.

## Abbreviations

Cy7MX	cyanine-7 methoxyamine
AuNP	gold nanoparticle
PEG	polyethylene glycol
AP	apurinic/apyrimidinic
PET	positron emission tomography
NIR	near infrared
BER	base excision repair
RES	reticuloendothelial system
EPR	enhanced permeability and retention
DLS	dynamic laser scattering
AAS	atomic absorption spectroscopy
GFAAS	graphite furnace atomic absorption spectroscopy
SDS	sodium dodecyl sulfate
DAPI	4′,6-diamidino-2-phenylindole
gold	Au
CHCl_3_	chloroform
DI H_2_O	deionized water
PBS	phosphate buffer saline
HAuCl_4_·3H_2_O	gold(III) chloride trihydrate
DDA	dodecylamine
Meo-PEG-SH	alpha-methoxy-omega-mercapto poly(ethylene glycol)
5-Fdu	5-fluorodeoxyuridine
UDG	uracil DNA glycosylase
HNO_3_	nitic acid
