# Supplementary material for: Gold Nanoparticle-Based Fluorescent Theranostics for Real-Time Image-Guided Assessment of DNA Damage and Repair
Source: Int J Mol Sci. 2019 Jan 22;20(3):471. doi: 10.3390/ijms20030471 (PMC6387448; doi:10.3390/ijms20030471)
Supplement: Supplementary file 1 [file ijms-20-00471-s001.pdf]

# Supplementary Materials

# Gold Nanoparticle-Based Fluorescent Theranostics for Real-time Image-Guided Assessment of DNA Damage and Repair

**Shriya S. Srinivasan[1], Rajesh Seenivasan[1], Allison Condie[2], Stanton L. Gerson[3], Yanming Wang[2,*], Clemens Burda[1,*]**

[1] Center for Chemical Dynamics and Nanomaterials Research, Department of Chemistry, Case Western Reserve University, 10900 Euclid Avenue, Cleveland, OH 44106 USA. *e-mail: burda@case.edu

[2] Department of Radiology, Case Western Reserve University, 10900 Euclid Avenue, Cleveland, OH 44106 USA. *e-mail: yxw91@case.edu

[3] Department of Hematology and Oncology, Case Comprehensive Cancer Center, Case Western Reserve University, Cleveland, OH 44106, USA.

* Correspondence: e-mail: burda@case.edu; Tel: (+1)-216-368-5313, yxw91@case.edu; Tel: (+1)-(216)-844-3288

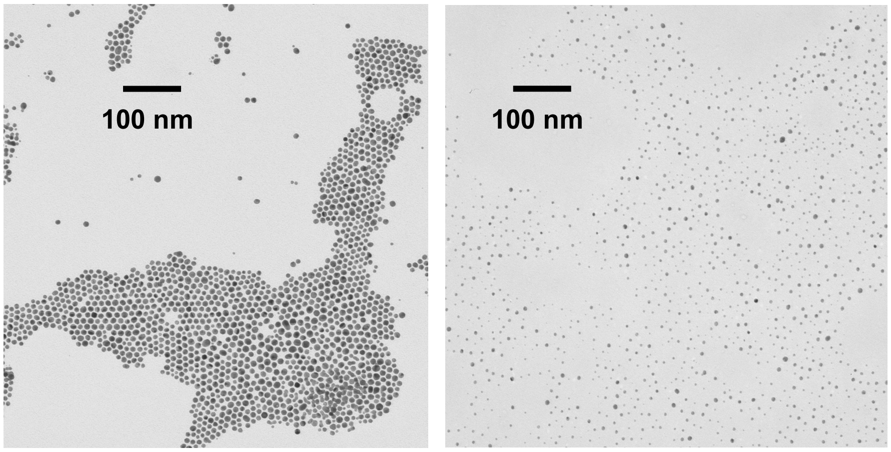


**Figure S1.** TEM image of AuNPs confirming the ligand exchange process and appropriate size dispersion (5 ± 2 nm) of particles. Left DDA capped Au NPs and right PEGylated Au NPs. DLS provides 32 nm hydrodynamic diameter.

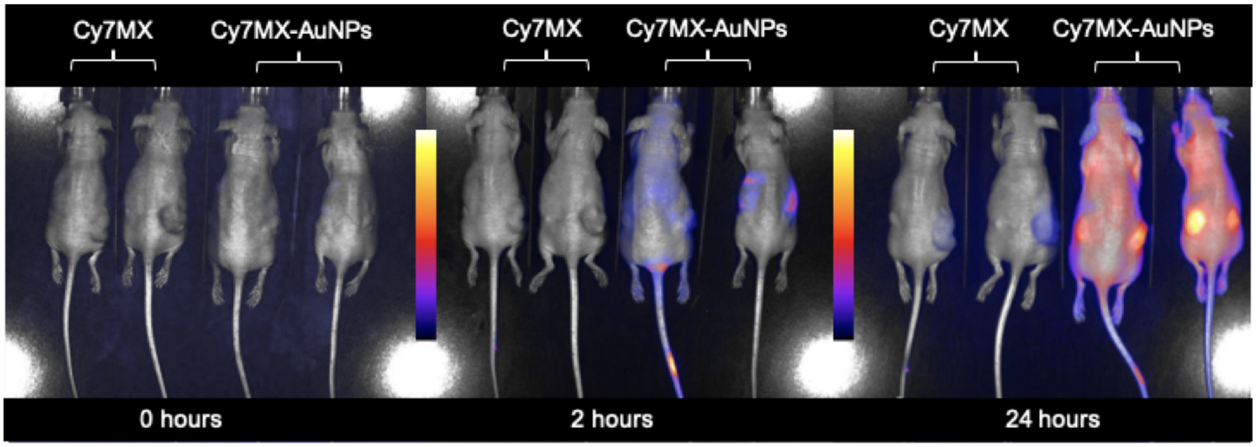


**Figure S2.** Fluorescence imaging of animals in the chemotherapy group treated with Cy7MX and Cy7MX-AuNPs indicates selective uptake by the tumors on the flank and increased circulation of the probe when delivered with the Au NP carrier. Representative images from 0, 2, and 24 hours are provided.
